# A Model of Bacterial Intestinal Infections in Drosophila melanogaster

**DOI:** 10.1371/journal.ppat.0030173

**Published:** 2007-11-23

**Authors:** Nadine T Nehme, Samuel Liégeois, Beatrix Kele, Philippe Giammarinaro, Elizabeth Pradel, Jules A Hoffmann, Jonathan J Ewbank, Dominique Ferrandon

**Affiliations:** 1 Equipe Fondation Recherche Médicale, UPR 9022 du CNRS, Institut de Biologie Moléculaire et Cellulaire du CNRS, Strasbourg, France; 2 Equipe Fondation Recherche Médicale, Centre d'Immunologie de Marseille-Luminy, Université de la Méditerranée, Case 906, Marseille, France; 3 INSERM U631, 13288 Marseille, France; 4 CNRS UMR6102, 13288 Marseille, France; Stanford University, United States of America

## Abstract

Serratia marcescens is an entomopathogenic bacterium that opportunistically infects a wide range of hosts, including humans. In a model of septic injury, if directly introduced into the body cavity of *Drosophila*, this pathogen is insensitive to the host's systemic immune response and kills flies in a day. We find that S. marcescens resistance to the *Drosophila immune deficiency* (*imd*)-mediated humoral response requires the bacterial lipopolysaccharide O-antigen. If ingested by *Drosophila*, bacteria cross the gut and penetrate the body cavity. During this passage, the bacteria can be observed within the cells of the intestinal epithelium. In such an oral infection model, the flies succumb to infection only after 6 days. We demonstrate that two complementary host defense mechanisms act together against such food-borne infection: an antimicrobial response in the intestine that is regulated by the *imd* pathway and phagocytosis by hemocytes of bacteria that have escaped into the hemolymph. Interestingly, bacteria present in the hemolymph elicit a systemic immune response only when phagocytosis is blocked. Our observations support a model wherein peptidoglycan fragments released during bacterial growth activate the *imd* pathway and do not back a proposed role for phagocytosis in the immune activation of the fat body. Thanks to the genetic tools available in both host and pathogen, the molecular dissection of the interactions between S. marcescens and *Drosophila* will provide a useful paradigm for deciphering intestinal pathogenesis.

## Author Summary

The gut is a crucial interface of the host with its environment and represents an important portal of entry for pathogens. Here, we have developed a novel model of intestinal infections in the genetic model organism Drosophila melanogaster using the potent entomopathogen bacterium Serratia marcescens. In contrast to other enteropathogens, this bacterium traverses the intestinal epithelium despite a local immune response and gains access to the body cavity of the fruit fly. The cellular arm of innate immunity controls its proliferation in the hemocoele. Interestingly, ingested bacteria that have moved to the hemolymph compartment are not detected by the humoral immune system of the fly unless phagocytosis is blocked. In a septic injury model, S. marcescens kills its host in a day. In contrast, the flies succumb slowly to an intestinal infection, even though the bacterium is present in the hemolymph. We surmise that the bacterium expresses distinct virulence programs according to the mode of infection. Thanks to the genetic tools available in both host and pathogen, the molecular dissection of the interactions between S. marcescens and *Drosophila* will provide a useful paradigm to decipher intestinal pathogenesis.

## Introduction

A major arm of the *Drosophila* host defense against microbial infections is the systemic humoral response that consists primarily of the massive synthesis and release of potent antimicrobial peptides (AMPs) by cells of the fat body (reviewed in [1,2]). The role of these AMPs in fighting infections has been previously demonstrated by biochemical and genetic assays [3,4]. The detection of invading microorganisms by host receptors of the Peptidoglycan recognition protein (PGRP) or Gram-negative binding protein (GNBP) families triggers the activation of signal transduction pathways in the fat body via the Toll and PGRP-LC receptors [5–12]. Genetic analysis has led to the delineation of two NF-κB-like signaling pathways, the *immune deficiency* (*imd*) and the *Toll* pathways, which control the expression of genes encoding the AMPs via activation of distinct NF-κB transcription factors. The *imd* pathway principally leads to the activation of Relish and transcription of genes mediating the response against Gram-negative bacteria. The *Toll* pathway, via activation of Dorsal-related immunity factor (DIF), controls the expression of effector genes that mainly target fungi and Gram-positive bacteria.

Barrier epithelia also produce AMPs. Their overall contribution to the *Drosophila* host defense had not been assessed, however, for lack of relevant infection models [13–15]. This situation has now changed thanks to studies on intestinal infection models. The intestine's function is to assimilate microbe-rich food such as decaying fruits while preserving the fly from infections. The midgut is protected by a chitinous physical barrier, the peritrophic matrix that lines the intestinal epithelium and contains microbes within the lumen of the digestive tract. This matrix is continuously synthesized by the cardia (also known as the proventriculus), an elaborate structure at the entrance of the midgut that can also express AMPs such as Diptericin [14]. This expression of AMPs is enhanced by activation of the *imd* pathway and provides a partial protection against entomopathogenic bacteria such as Pseudomonas entomophila [16]. It complements another arm of the immune response to intestinal infections, namely the production of reactive oxygen species (ROS), and is important since some yeasts and bacteria are resistant to host ROS [16,17]. To date, all studies on the intestinal immune response have been performed with bacteria that consistently remain confined to the digestive tract, whether entomopathogenic (P. entomophila) or phytopathogenic (*Erwinia carotovara carotovora*) [18,19]. Bacteria present in the digestive tract induce the *imd* pathway locally in the cardia and systemically in the fat body of larvae (P. entomophila, E. carotovora) and adults (P. entomophila) [16,18,19]. These bacteria are thought to release small peptidoglycan (PGN) fragments that pass the intestinal epithelium and bind to the membrane-bound PGRP-LC and hemolymphatic PGRP-LE microbial receptors that in turn activate the *imd* pathway in fat body cells [20]. At the same time, the system appears to be negatively regulated by the amidase PGRP-LB, which can break down PGN fragments into nonstimulatory molecules. Thus, the ingestion of a small PGN fragment, tracheal cytotoxin, triggers the *imd* pathway and the systemic immune response only in *PGRP-LB* mutant flies. Further, the response to ingested tracheal cytotoxin is blocked when recombinant PGRP-LB is injected directly into the hemolymph of the *PGRP-LB* mutants [20].

Some pathogens have developed strategies that allow them to gain access to target host tissues and that help them survive the host immune response. We have chosen to investigate the interactions between *Drosophila* and a potent entomopathogen, Serratia marcescens, as a model for understanding how flies handle invasive pathogens. This enterobacterium is a pathogen for many other host organisms, including plants and nematodes and is also an opportunistic pathogen of mammals ([21,22] and references therein). As regards human health, S. marcescens is increasingly responsible for nosocomial infections in intensive care and neonatal units and it commonly infects people suffering from chronic granulomatous disease [23]. It was also the contaminant that led to the recent withdrawal of an influenza vaccine [24]. In the present study we have used S. marcescens Db11, a streptomycin-resistant derivative of a strain originally isolated by Flyg and Boman from moribund flies [25,26]. Db11 is virulent when inoculated (septic injury model), but is much less virulent when fed to the flies (ingestion model). Genetic studies with S. marcescens have led to the isolation and identification of two bacterial strains that are almost avirulent in *Drosophila* after septic injury, Db1140 and 20C2. Db1140 has pleiotropic defects compared to its Db11 parent: the activity of secreted proteases is strongly decreased [27]; it produces a truncated lipopolysaccharide (LPS) lacking the O-antigen [22], and is nonmotile [28]. The 20C2 transposon insertion mutant also lacks the LPS O-antigen, but secretes proteases and is motile [22]. Both strains are highly attenuated in a Caenorhabditis elegans model of infection [22].

We have examined here the distinct pathogenic properties that S. marcescens displays upon ingestion as opposed to direct injection into *Drosophila*. We show that injected S. marcescens resists the systemic immune response essentially because of the O-antigen of its LPS. We find that ingested S. marcescens escapes from the digestive tract into the hemocoele and document its passage through the intestinal epithelium. Although the *imd* pathway does not protect the fly effectively against Db11 in the septic injury model, we found that S. marcescens is sensitive to the *imd* pathway–mediated local response in the gut. We demonstrate that phagocytosis is an effective defense against ingested S. marcescens that have escaped into the hemolymph but not against Db11 introduced directly into the hemocoele. Finally, we investigate why ingested Db11 does not induce systemically the *imd* pathway when present in the hemolymph.

## Results

### S. marcescens Is Resistant to the Systemic Immune Response in the Septic Injury Model

**Figure 1:**
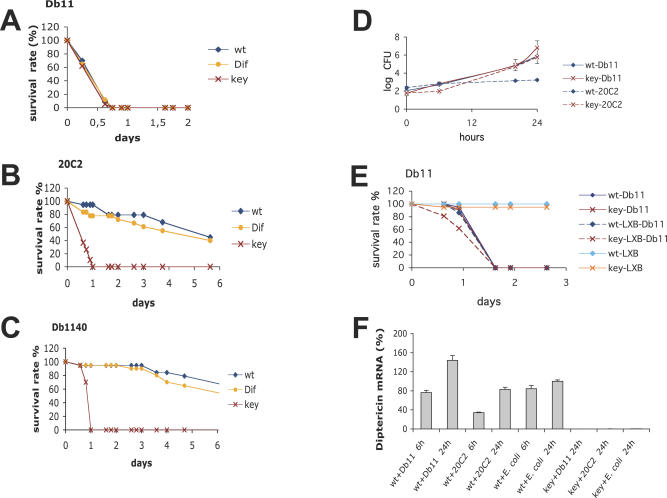
S. marcescens Is Resistant to the Humoral Immune Response in the Septic Injury Model (A–C) Wild-type (wt), *Dif*, or *key* flies were pricked using a thin needle dipped in a diluted overnight culture of Db11 (A), 20C2 (B), or Db1140 (C) (OD_600_ = 0.1) to introduce about 100 bacteria per fly. Flies were kept at 25 °C. Survival was monitored and expressed in % of surviving flies. Note that 20C2 and Db1140 kill *key* flies while they are less virulent in wild-type or *Dif* flies. (D) The hemolymph of pricked flies incubated was collected and spread on agar plates containing the appropriate antibiotic. The experiment was performed at 20 °C to insure that we would obtain enough surviving flies at the late time points of the experiment. 20C2 bacteria are kept in check in the hemolymph of wild-type, but not *key* flies. CFU: colony-forming units (logarithmic scale). (E) Flies were either preinjected with latex beads (LXB) or nontreated and then submitted to an immune challenge with Db11, except for nonchallenged controls. Survival was monitored at 20 °C. (F) *Diptericin* expression was induced in wild-type flies at 20 °C after pricking with Db11 or 20C2 but not in *key* mutant flies. *Diptericin* mRNA levels were measured by quantitative RT-PCR. The expression induced 24 h after an E. coli septic injury is taken as a reference. *Dif* flies exhibited normal induction of *Diptericin* after S. marcescens septic injury (not shown).

Previous studies have indicated that S. marcescens Db11 is highly pathogenic in a *Drosophila* septic injury model [22,27]. We address here the molecular basis of S. marcescens virulence in this system and determine whether the classical arms of *Drosophila* host defense, namely the humoral and cellular responses, are efficient in fighting off this bacterium. As shown in Figure 1A, wild-type, *Toll* (*Dif*), or *imd* (*kenny* [*key*]) pathway mutant flies died at the same rate, in less than a day, after a challenge with about 100 Db11 bacteria. We also did not observe a difference between *key* and wild-type flies after a challenge with a lower dose of about five bacteria (unpublished data). The similar sensitivity of wild-type and mutant flies to Db11 was mirrored by the rate of bacterial growth in the hemolymph of infected flies (Figure 1D). These data suggest that strain Db11 is resistant to the insect systemic immune response since immunodeficient flies do not display an enhanced susceptibility to Db11. We next saturated phagocytosis in wild-type flies by injecting latex beads that are taken up, but not degraded, by hemocytes, thus effectively blocking this cellular defense mechanism [29,30]. We observed a similar death rate between latex bead–injected and noninjected flies after a Db11 challenge, indicating that the cellular immune response does not contribute significantly to host defense against this bacterium (Figure 1E).

One possible explanation for the virulence of S. marcescens is that this pathogen inhibits the activation of the *Drosophila* systemic immune response. This is not the case, however, because we detected the induction of the *Diptericin* gene, a classical readout of *imd* pathway activation, upon a challenge with the wild-type or mutant S. marcescens strains Db11, 20C2 (Figure 1F), and Db1140 (unpublished data) even though only about 100 bacteria were introduced into the insect body cavity. The expression of another *imd*-dependent AMP gene, *CecropinA*, was similarly induced (unpublished data). Thus, the virulence of S. marcescens in the septic injury model is likely due to its ability to withstand the systemic immune response mediated by the *imd* pathway. Indeed, the stimulation of the systemic immune response either by a prior challenge with Escherichia coli or Enterobacter cloacae, or the ubiquitous overexpression of *Diptericin*, did not confer any protection to the flies against a subsequent challenge with Db11 (unpublished data and Figure S1A). In keeping with these observations, S. marcescens Db11 was also resistant to the action of 200 μM Drosocin, Cecropin, and Defensin in an in vitro assay (D. Rabel, personal communication), even though at such a high concentration these AMPs are highly effective against several other bacteria [3].

We next analyzed the pathogenicity of S. marcescens using bacterial mutants with an attenuated virulence. We have recovered several mutants that disrupt an operon involved in O-antigen biosynthesis from screens performed in C. elegans [22,31]. The 20C2 mutant was susceptible to the *imd*-dependent systemic immune response: *key* but not *Dif* or wild-type flies succumbed to 20C2 infection within 24 h (Figure 1B). Accordingly, 20C2 bacteria grew rapidly only in the *key* mutant background (Figure 1D). Nevertheless, this bacterial mutant ultimately killed 50% of the wild-type flies in approximately 5 d. Similar results were obtained with other O-antigen-deficient mutants bearing different disruptions of the same operon (unpublished data). We also tested the Db1140 strain, which also lacks a LPS O-antigen, and found that it behaves like 20C2 in the septic injury model both in wild-type and *imd* pathway mutant flies.

### S. marcescens Can Infect *Drosophila* through the Digestive Tract

**Figure 2:**
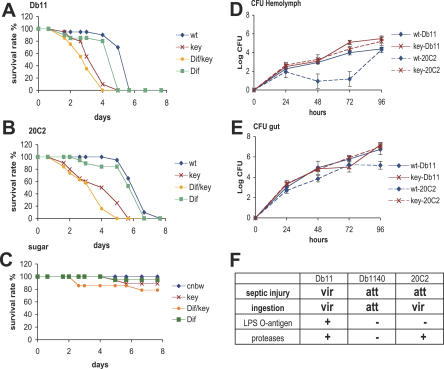
Ingested S. marcescens Db11 Kills *Drosophila* Flies Slowly (A–C) Db11 (A) or 20C2 (B) bacteria were fed to wild-type (wt) or mutant flies at 25 °C. Survival is expressed in % of surviving flies. (A) median survival times in days (LT50): wt: 5.7; *Dif*: 5.0; *key*: 4.0; *Dif*. *key*: 3.0 (the difference between wt and all mutants is significant (*p* < 0.0001)); (B) wt: 6.7; *Dif*: 6.7; *key*: 4.5; *Dif*, *key*: 4.0 (the difference between wt and *key* is significant (*p* < 0.0001)). LPS-defective 20C2 kills wild-type flies more slowly than Db11: LT50 for flies feeding on 20C2 is 6.7 d compared to 5.7 d for flies on Db11 (*p* < 0.0001). (C) Flies were fed on a 50-mM sugar solution as control. All three experiments were performed in parallel. (D) The hemolymph of batches of 20 infected flies was collected and plated on LB agar containing the appropriate antibiotics. CFU: colony-forming units (logarithmic scale). The values for the 72-h time point are as follows: wt-Db11-GFP: 4.0 +/-0.26 (standard deviation); *key*-Db11-GFP: 5.1 +/-0.21; wt-20C2-GFP: 1.18 +/- 0.8 ; *key*-20C2-GFP: 4.4 +/- 0.3 (*n* = 4 experiments). (E) Guts were dissected and crushed, and dilutions of the extracts were plated as in (D). Note that flies are continuously feeding on a bacteria-containing sucrose solution. (F) Summary of the properties of the S. marcescens strains used in this study. Vir: virulent; att: attenuated virulence.

*Serratia* is commonly found in the environment and contaminates insects in the absence of injury in insectaries [21,32]. We reestablished a model of infection through an oral route [25]. We observed that wild-type flies feeding continuously on Db11 diluted in a sucrose solution were killed within 6 d (Figure 2A). We found that the death rate of these infected flies varied with the bacterial load in the food solution and with temperature (Figure S2 and unpublished data), whereas the death rate was negligible in flies feeding on sugar solution alone (Figure 2C). Flies do not succumb to a secondary infection by bacterial commensals of the midgut, because flies feeding on Db11GFP (a derivative of Db11 that expresses GFP and is resistant to ampicillin and streptomycin) in the presence of ampicillin and streptomycin died at the same rate as control flies fed on Db11GFP alone (unpublished data). The slow killing rate of Db11, as compared to that observed in the septic injury model, is not due to the containment of Db11 within the digestive tract, since we were able to recover bacteria from the hemolymph of infected flies (Figure 2D). The bacterial titer in the hemolymph increased slowly, as did the bacterial titer in the gut (Figure 2E). These data suggest that Db11 can rapidly escape from the digestive tract into the internal body cavity of the host, yet fails to kill it rapidly.

**Figure 3:**
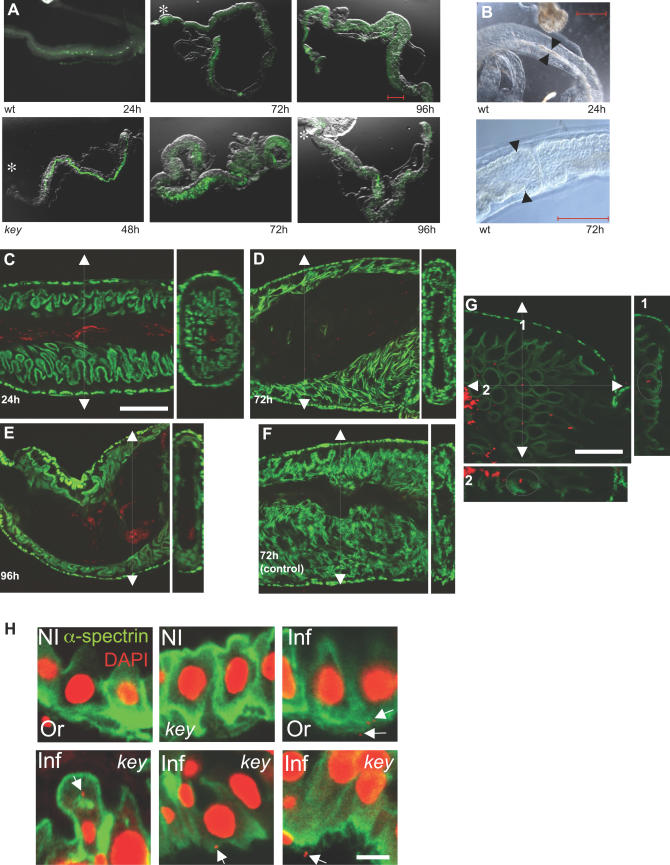
Effect of S. marcescens Oral Infection on the Structure of the Midgut (A) S. marcescens Db11-GFP is found throughout the digestive tract after ingestion in both wild-type (upper panels) and *key* flies (lower panels), as observed with a dissecting microscope equipped with epifluorescent illumination. The fluorescent image (FITC channel) has been superimposed onto an oblique transmitted-light brightfield picture (except for the top left panel). Anterior is to the left. The cardia (proventriculus) is indicated by an asterisk. Scale bar is 150 μm. (B) The bacteria distend the gut lumen (indicated by arrowheads) during the later stages of the infection. Note the thinning of the epithelium in places where the digestive tract is bloated. Scale bar is 150 μm. Intestines of insects feeding on Db11 for 24 h are indistinguishable from those of control insects fed on sugar solution. (C–G) Confocal optical sections from the anterior part of the midgut of insects feeding on Db11-DsRed, after fixation and FITC-labeled phalloidin staining, that reveals the actin cytoskeleton enriched in the apical region of gut cells. Left panels show a longitudinal section and right panels show the transverse section from the axis displayed in each left panel. Note the thinning of the intestinal epithelium after 72 h (D) or 96 h (E) of infection as compared to the control feeding on sucrose (F) or after 24 h (C) of infection (OD = 0.1). (C–G) Scale bars are 50 μm. (G) Db11-DsRed infection at higher concentration (OD = 0.5) observed after 48 h: a bacterium inside a cell is shown at the center of the main panel and is indicated by circles in the lateral sections. (H) Immunostainings on frozen sections from the midgut of wild-type Oregon (Or) or *key* mutant flies fed for 6 h on sucrose (NI: noninfected) or OD = 0.2 Db11-DsRed (Inf: infected). The α-spectrin antibody staining (green) is enriched at the cell periphery. Nucleic acids of the epithelial cell and bacteria (arrows) is shown in red (DAPI staining). Scale bar is 10 μm.

**Figure 4:**
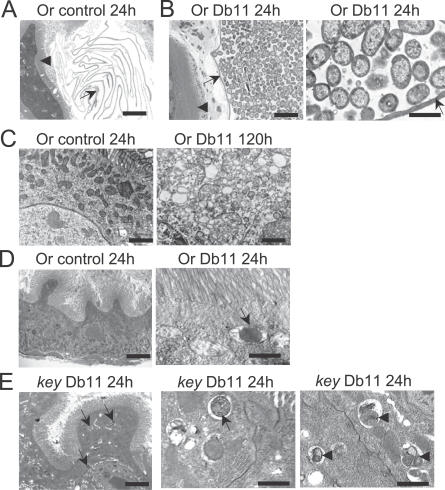
S. marcescens Is Found within Intracellular Vacuoles in the Midgut Epithelium Ultrastructure of wild-type and *key* mutant midguts fed on Db11 (OD = 0.1) or sucrose. (A, B) The midgut epithelium with associated microvilli (arrowhead) is separated from the gut lumen by the peritrophic membrane (arrow). (A) Oregon fly fed for 24 h on sucrose. Note that the peritrophic membrane is sometimes folded extensively and that no microorganisms are present. (B) Oregon fly fed for 24 h on Db11. The bacteria are confined inside of the peritrophic membrane and are shown at high resolution in the right panel. Scale bars: (A, B) left panel: 5 μm; (B) right panel: 1 μm. (C) Oregon fly fed for 24 h on sucrose (left panel) or for 120 h on Db11 (right panel). The infected midgut epithelial cell displays translucent vacuoles evocative of cellular stress. (D) Oregon fly fed for 24 h on sucrose (left panel) or for 24 h on Db11 (right panel). An intracellular bacterium inside a vacuole is indicated by an arrow. (E) *key* mutant fly fed for 24 h on Db11. Intracellular bacteria are indicated by arrows and putative degraded bacteria by arrowheads. Scale bars are 5 μm for low magnification pictures ([A], [C], left panel in [B], [D], and [E]) and 1 μm in high magnification pictures (right panels in [B], [D], and [E]).

We then examined the infectious process by microscopy using GFP- or DsRed- labeled bacteria, concentrating initially on the first 2 d of the infection. We established the presence of S. marcescens along the whole length of the digestive tract (Figure 3A). During the initial stage of the infection, midgut morphology appeared normal when observed at low magnification, with bacteria mainly confined to the lumen. We could observe bacteria penetrating the deep invaginations of the acid-secreting copper cells [33] after 48 h of infection (Figure S3). Even though the bacteria remain topologically outside of the epithelial layer, they have nevertheless crossed the peritrophic matrix during this period. Although the integrity of the peritrophic matrix appeared preserved at the ultrastructural level in our ultrathin sections (Figure 4A and 4B), we cannot exclude that it is locally ruptured. Once they had crossed the intestinal epithelium, bacteria were sometimes observed in the midgut muscles and surrounding tracheoles (Figure S4).

To determine whether Db11 pass through or in between intestinal epithelial cells, we used higher concentrations of bacteria and observed the location of Db11 in fixed tissues either as whole mount preparations or on frozen sections. We occasionally observed bacteria in the anterior midgut and cardia that appeared to be intracellular in whole mount preparations (Figure 3G). More often, we observed bacteria localized close to the apical parts of the epithelial cells (Figure S5) or even entering these cells (Figure S5D). In frozen sections, we relied on DAPI staining, as DsRed fluorescence was often strongly reduced. Figure 3H shows the intracellular distribution of bacteria in wild-type (1.83 bacteria on average per section) and *key* mutants (4.29 bacteria on average per section).

We seldom observed bacteria crossing the intestinal barrier in wild-type flies by electron microscopy. In the rare cases where we did detect bacteria within the epithelium during early infection, the bacteria were detected at an intracellular location, within a vacuole (Figure 4D). To increase the number of observed events, we analyzed immunocompromised *imd* pathway mutant flies (*key*), since they display a higher number of intracellular Db11 (Figure 3H). We detected many vacuoles that evoked autophagic bodies (Figure 4E) in intestinal cells. Other vacuoles enclosed bacteria. These data establish that S. marcescens can invade intestinal epithelial cells.

By 72 h of infection, the digestive tract was distorted with more bacteria concentrated in the lumen, which was dilated in some places (Figure 3A and 3B). At this stage of the infection, the structure of the midgut began to be affected, and the epithelial lining appeared very thin (Figure 3B; compare Figure 3C and 3E; 3D and 3F). The alteration of the midgut epithelium was obvious at the ultrastructural level: the cytoplasm of intestinal cells appeared very different from that of control epithelial cells (Figure 4C). It was characterized by the presence of numerous small electron-translucent vacuoles, as noted previously in *Shigella*-infected Henle cells, indicating an important cellular stress [34]. The cells did not usually exhibit any of the hallmarks of impending apoptosis, such as nuclear fragmentation or a homogenous cytosol with a low number of organites, nor of necrosis.

We reasoned that bacterial proteases might be responsible for the progressive degradation of gut structure during infection. We therefore tested the Db1140 strain, which is partially deficient in protease activity, and possibly for other functions [27]. This strain is sensitive to the *imd*-dependent systemic response because, like 20C2, it lacks the LPS O-antigen (Figures 1C and 2F). In the ingestion model, Db1140 failed to kill wild-type flies (Figure 7D), unlike Db11 and 20C2 (Figure 2). We observed that gut integrity was preserved in flies that were fed on Db1140 (Figure S6). Yet, we found in rare instances that these bacteria still appeared to be taken up by intestinal cells (Figure S6B, arrow). In addition, Db1140 was transiently recovered from the hemolymph in immunodeficient flies, which slowly succumbed to the infection (Figure 7G). Taken together, these data suggest that the degradation of the gut contributes to the lethal outcome of the oral infection.

### *imd* Pathway Mutants Are More Susceptible to S. marcescens Oral Infection than Wild-Type Flies

To delineate the host response to intestinal infections, we first determined whether the *Drosophila* signal transduction pathways regulating the systemic immune response to septic injury are also involved in the host defense in our ingestion model. We found that *Dif* mutant flies [35], in which induction of *Toll* pathway target genes is impaired, displayed an increased susceptibility to oral infections with S. marcescens Db11 (median survival time to death [LT50] 5.0 d) when compared to wild-type flies (LT50: 5.7 d) (Figure 2A and 2B). However, the susceptibility to the ingestion of Db11 was more pronounced with *key* (*kenny*) mutants (LT50: 4.0 d) in which the *imd* pathway is defective [36], even though Db11 is resistant to the *key*-dependent systemic immune response in the septic injury model. Similar results were found with other mutants of the *imd* pathway, including *imd*, *Relish*, *DREDD*, and *FADD*, while *PGRP-LC* and *PGRP-LE; PGRP-LC* mutants exhibited a mild phenotype (Figure S7 and unpublished data). The sensitivity of *key* mutants was correlated at 72 h post-infection with a reproducible 10-fold increase in the number of bacteria retrieved from the hemolymph, as compared to wild-type (Figure 2D). *key* mutants displayed an altered epithelial midgut morphology 24 h earlier than wild-type flies (Figure 3A), suggesting that the *imd* pathway helps control the deleterious effects of Db11 in the midgut, even though there was no difference between the number of Db11 bacteria recovered from *key* mutant and wild-type midguts (Figure 2E), possibly because the flies constantly feed on fresh bacteria.

To determine whether the *imd* pathway is involved in the host response to intestinal infections, we next analyzed the behavior of mutant bacteria that are sensitive to the action of the *imd* pathway in the septic injury model (Figure 1). Wild-type flies feeding on 20C2 bacteria died with a 1-d delay as compared to Db11 (LT50: 6.7 d; Figure 2B). 20C2 bacteria were detected only transiently in the hemolymph at 24 h (before increasing again from 72 h onwards), suggesting that they are also susceptible to the host response to intestinal infections (Figure 2D). As in the septic injury model, 20C2 was, however, retrieved in large amounts in the hemolymph of *key* mutant flies (Figures 1D and 2D), indicating that the *imd* pathway either controls the growth of the LPS-defective 20C2 in the hemocoele or hinders its escape from the gut.

**Figure 5:**
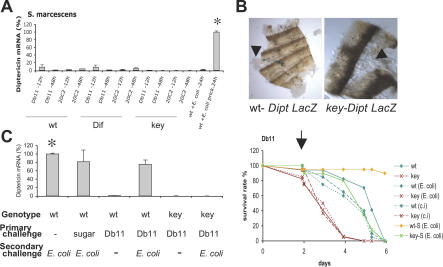
The Systemic Humoral Immune Response Is Not Triggered by Ingested S. marcescens (A) *Diptericin* mRNA levels were measured by quantitative RT-PCR. No significant expression was induced after feeding with S. marcescens Db11 or 20C2, or with nonpathogenic *E. coli 1106* (wt, *Dif*, *key*, and wt E. coli samples, respectively). The expression induced by an *E. coli 1106* septic injury is taken as a reference (*). No induction of *Cecropin* expression could be detected; the systemic immune response was not significantly induced at later time points (up to 144 h; unpublished data). (B) No expression of ß-galactosidase was detected in fat body cells of *Diptericin*-*lacZ* transgenic flies as indicated by the absence of blue staining in fat body lobules (arrowheads). The expression observed in wild-type flies is in pericardial cells and is also found in *key* mutants, although they are absent in this dissection. (C) Flies that have fed on Db11 for 48 h (primary challenge) are still able to mount a systemic immune response when pricked by either E. coli (secondary challenge: 6 h after injury) or Db11 (not shown). This response is dependent on the *imd* pathway as *key* mutants showed no response. *Diptericin* mRNA levels were measured by quantitative RT-PCR 54 h after the beginning of bacterial ingestion. *: 100 % reference: wild-type flies challenged with E. coli for 6 h. (D) Flies fed on Db11 are still able to mount an effective immune response since they do not succumb to a secondary *E. coli 1106* septic injury (wt and *key* (E. coli)) at an enhanced rate, as compared to mock-challenged flies (wt and *key* (ci); ci: clean injury). The arrow indicates the time point (day 2) at which the secondary challenge was performed (all curves correspond to flies that were feeding on Db11, except for the solid yellow and green lines, which correspond respectively to control wt and *key* flies fed on sugar [S] solution and challenged with E. coli at day 2). wt and *key* flies feeding on Db11 and not challenged secondarily by E. coli are also shown (wt and *key*).

Because a systemic response in the fat body is triggered by some intestinal pathogens [16,37], we suspected that that it might account for the enhanced susceptibility of *imd* pathway mutants to Db11 oral infections. We therefore measured this response in flies that have ingested Db11 or 20C2 and detected no induction of the *imd*-dependent AMP genes *Diptericin* and *Cecropin* in whole flies (Figure 5A and unpublished data). We also observed no induction of AMP genes in the fat body using a set of GFP reporter genes or a *Diptericin-lacZ* transgene (Figure 5B and unpublished data). This lack of a systemic response is not due to an active inhibition by ingested S. marcescens since an additional septic injury by E. coli led to a normal induction of *Diptericin* expression (Figure 5C and unpublished data). Flies that had ingested Db11 also resisted a septic wound with E. coli as robustly as flies feeding on sugar solution alone (Figure 5D). Altogether, our experiments revealed that ingested Db11 (and 20C2) fail to elicit a response when they gain access into the hemolymph from the gut.

### S. marcescens Is Sensitive to the *imd*-Dependent Local Immune Response in the Midgut

**Figure 6:**
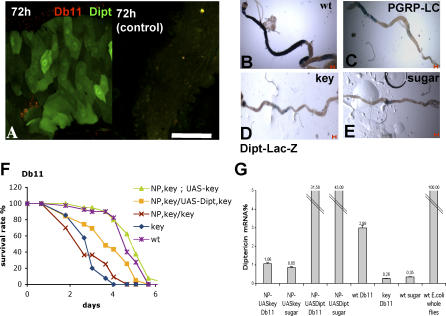
The *imd*-Dependent Immune Response in the Midgut Mediates Host Defense against S. marcescens Intestinal Infections (A) A *Diptericin-GFP* transgene is induced in the posterior part of the midgut following an oral challenge with S. marcescens Db11-DsRed (OD = 0, 1, 72 h), as observed by confocal microscopy. Scale bar is 50 μm. (B–E) The expression of *Diptericin* in the midgut was visualized in dissected preparations of flies carrying a *Diptericin-lacZ* reporter transgene that have fed on Db11 (B). This expression is reduced in the *imd*-pathway mutants *PGRP-LC* (C) and absent in *key* (D) midguts. The transgene is not induced in flies feeding on sugar solution (E). Scale bar is 150 μm. (F) *key* mutant flies expressing a *Diptericin* transgene in the midgut under the control of the *NP3084 Gal4* driver (NP3084-*Gal4, key/key, UAS-Diptericin)* survive longer than *key* mutants following an oral infection at 29 °C with Db11 (median survival time [LT50]: 3.7 versus 3.0; *p* < 0.0001). The rescue of the *key* mutant phenotype by the expression of a *key* transgene (NP3084-*Gal4, key/key; UAS-key)* in the midgut is complete (LT50: 5.1, *p* < 0.0001). Similar results have been obtained with two other Gal4 drivers that induce the expression of UAS-transgenes in the midgut. Experiments have been repeated twice. NP3084-*Gal4, key/key* flies die at the same rate as *key* mutant flies (LT50 2.7 versus 3.0, *p* = 0.47). (G) Levels of *Diptericin* transcripts in midgut of wild-type and transgenic flies as measured by quantitative real-time RT-PCR. The normalized signal (using ribosomal protein 49 gene transcript) obtained from extracts of whole flies undergoing a systemic immune response has been arbitrarily set at 100%. The measured values are displayed on the top of each bar. The overexpression of *Diptericin* induced by the *UAS-Dipt* transgene (32% and 43% for flies fed with sugar and Db11 [Db11] or sugar alone [sugar]) is much higher than that observed in wild-type flies infected *per os* (3%).

In the absence of a systemic response, we searched for a local immune response in the midgut to account for the role of the *imd* pathway in host defense. We found that *Diptericin* transcription was induced in large segments of the midgut by uptake of *S. marcescens*, as judged by the expression of *lacZ* and GFP reporter genes (Figure 6A, 6B, and 6E). Importantly, *Diptericin* expression was reduced in *PGRP-LC* mutants and absent in *key* mutants, indicating that the *imd* pathway is involved in the inducible expression of this AMP in the gut (Figure 6C and 6D). Among several AMP reporter genes tested (Table S1), *Diptericin-GFP* was the only one that displayed a Db11-induced expression in the digestive tract. We observed, however, the induction of other AMP reporter genes in various epithelial tissues (Table S1).

**Figure 7:**
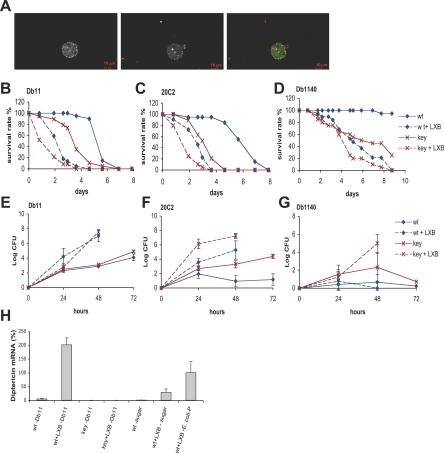
Phagocytosis Plays an Essential Role in the Control of Intestinal S. marcescens Infection (A) A *pTEP1*-*GFP*-expressing hemocyte that has phagocytosed Db11-DsRed bacteria. The picture was taken with the apotome microscope: left-panel (GFP); central panel (DsRed); right panel (merge). *pTEP1*-*GFP* expression specifically labels hemocytes. Bacteria that have been engulfed appear yellow in the right panel. (B–D) Phagocytosis inactivation through the injection of latex beads (+LXB) leads to the earlier demise of treated flies as compared to control (untreated flies). (B) Db11: median survival time (LT50 in days), wt: 5.63, wt+LXB: 2.63, *key*: 3.63, *key* +LXB: 1.92 (LXB-treated versus untreated flies: *p* < 0.0001; wt+LXB versus *key* +LXB: *p* = 0.0089). (C) 20C2: wt: 6.88, wt+LXB: 2.63, *key*: 3.63, *key* +LXB: 1.292 (LXB-treated versus untreated flies: *p* < 0.0001; wt+LXB versus *key* +LXB: *p* = 0.0045). Db1140 (D). Latex bead–injected flies usually survive the procedure well when fed on sucrose alone (not shown). (E–G) The hemolymph of latex bead-injected and nontreated surviving flies (batches of 20 flies) that had been feeding on Db11-GFP (E), 20C2-GFP (F), Db1140-GFP (G) was collected and plated on agar plates containing the appropriate antibiotics. The counts were performed only up to 48 h in the case of LXB-treated flies since most were dead by 72 h. CFU: colony-forming units (log scale). (H) *Diptericin* mRNA levels were measured by quantitative RT-PCR in whole flies after Db11 feeding or LXB injection and Db11 feeding. A strong expression was induced only after phagocytosis inactivation through the prior injection of LXB. The expression induced by an *E. coli 1106* septic injury is taken as a reference.

We next set out to determine whether this local activation of the *imd* pathway in the cardia and the midgut is sufficient to confer some protection against ingested Db11. As shown in Figure 6F, *key* flies that expressed in the midgut either the *Diptericin* gene or the wild-type copy of *key*, displayed respectively a partially or totally rescued phenotype of survival to ingested Db11, as compared to *key* and wild-type flies. Note that the midgut Gal4 drivers we used for these experiments (NP1, NP3084) led to a level of *Diptericin* expression that was ten times higher than that observed during a Db11 oral infection in wild-type flies (Figure 6G). In keeping with a role for the *imd* pathway in the midgut, overexpression of *Diptericin* alone in wild-type flies increased resistance to intestinal Db11 infection (Figure S1B). Together, these experiments confirmed the antimicrobial capacity of *Drosophila* Diptericin in vivo, which had not been revealed in the context of the systemic immune response (Figure S1A) [4,16].

### Phagocytosis Plays an Essential Defense Role by Eliminating Bacteria That Have Crossed the Gut Epithelium

Db11 has the ability to traverse the gut barrier and yet does not induce a systemic immune response. Since these bacteria were observed to be either free in the hemolymph, or attached to or engulfed by hemocytes (Figure 7A), we evaluated the contribution of phagocytosis to the host defense in this model of infection using the latex bead saturation technique. Latex bead–injected flies showed a markedly enhanced susceptibility when fed Db11, 20C2, or Db1140 (Figure 7B–7D). This is unlikely to be due to a nonspecific effect of the latex beads since injection of cytochalasin D, which blocks phagocytosis, also leads to enhanced sensitivity to Db11 oral infection (Figure S8). The premature death of infected latex bead–injected flies correlated with the presence of a number of Db11 and 20C2 bacteria in the hemolymph that was almost two orders of magnitude higher than that in nontreated flies (Compare Figure 7E and 7F to Figure 2D). In contrast to Db11 and 20C2, Db1140 bacteria fail to kill their host, and few Db1140 bacteria were retrieved from the hemolymph of nontreated wild-type flies (Figure 7G). In contrast, *key* mutants in which phagocytosis had been blocked beforehand were killed by Db1140 and many bacteria where detected in the hemolymph (Figure 7G). Taken together, these data indicate that phagocytosis plays a vital role in the control of ingested S. marcescens that have gained access to the hemolymph. In the absence of this cellular immune response, the apparent proliferation of bacteria correlated with the advent of a strong systemic immune response as observed in latex bead–injected flies (Figure 7H).

### Is the Absence of a Systemic Response in the Oral Infection Assay due to a Reduced Proliferation of Bacteria in the Hemolymph?

**Figure 8:**
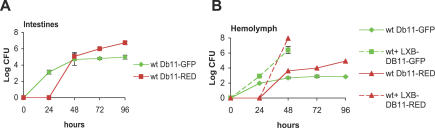
The Increasing Number of Db11 in the Hemolymph Results from Bacterial Passage from the Gut (A, B) Flies were fed for 24 h on Db11-GFP bacteria and then transferred to a vial containing only Db11-DsRed bacteria. The number of GFP- and DsRed-expressing bacteria was counted in the intestine (A) and in the hemolymph of latex bead–injected and nontreated surviving flies (batches of 20 flies) (B). The counts were performed only up to 48 h in the case of LXB-treated flies since most were dead by 72 h. CFU: colony-forming units (log scale).

At late stages of the infection, more than 1,500 bacteria could be retrieved from the hemolymph of a single fly, yet no systemic induction of the *imd* pathway could be detected. This lack of a response is not due to a number of bacteria that would be below a detection threshold since the injection of about 1,500 E. coli bacteria is sufficient to trigger a strong expression of *Diptericin* (unpublished data). Because the PGRP-LC/PGRP-LE sensing system is able to detect small PGN fragments released during bacterial growth and division [11,38], we wondered whether the bacteria that have traversed the intestine proliferate in the hemocoele. Indeed, the slow accumulation of S. marcescens in the hemolymph of flies feeding on Db11 could be due to bacterial proliferation. A second hypothesis is that the increase might be due to the continuous passage of bacteria from the intestine to the hemolymph. To discriminate between these possibilities, we first fed flies on Db11-GFP bacteria for 24 h. We then switched the flies to a food source that contained only Db11-DsRed. We monitored the number of green and red bacteria recovered both from the gut and from the hemolymph of infected flies (Figure 8A and 8B). As expected, the number of green Db11 in the digestive tract was overtaken by that of red Db11 bacteria (Figure 8A). Yet, the number of green bacteria did not decrease but remained stable. Strikingly, the count of green bacteria present in the hemolymph remained stable, whereas there was a steady increase in the number of Db11-DsRed (Figure 8B), thus indicating that the net increase of the number of bacteria in the hemolymph during intestinal infection is mostly due to bacteria that have traversed the digestive tract. These data, however, do not exclude the possibility of an equilibrium in the hemolymphatic compartment between bacterial proliferation on the one hand and phagocytosis on the other. Consistent with this hypothesis, we found that both GFP and DsRed-labelled bacteria numbers increased by several orders of magnitude within 48 h when we repeated this experiment in flies that had been previously injected with latex beads to saturate their phagocytic apparatus (Figure 8B). Two hypotheses can account for this result. One possibility is that the observed number of bacteria reflects a high rate of passage from the gut that is effectively counterbalanced by the cellular response when not inhibited by latex beads. The alternative is that this high number of bacteria results from bacterial division within the hemocoele that is not controlled when phagocytosis is blocked. To test these possibilities, we blocked the intestinal supply by feeding flies on a sucrose solution containing gentamicin following 1 d (or 4 h) of feeding on Db11. The treatment was effective in killing Db11 in the gut (Table S2 and unpublished data). In the treated flies, hardly any bacteria were recovered from the hemolymph. The decreased bacterial concentration in the hemolymph of gentamicin-treated flies is not the result of antibiotics treatment but of elimination of these bacteria by phagocytes, because we detected a large hemolymphatic bacterial count in gentamicin-treated flies in which phagocytosis had been blocked by latex beads injection (Table S2 and unpublished data). Importantly, this latter experiment in latex bead–injected flies shows that bacterial proliferation occurs in the hemolymph when phagocytosis is blocked and the intestinal reservoir depleted by antibiotics treament. Taken together, these data indicate that the high number of bacteria retrieved from the hemolymph of flies in which phagocytosis has been blocked results from bacterial division, which thus correlates with the elicitation of the *imd* pathway in the fat body.

## Discussion

In the septic injury model, S. marcescens is a potent pathogen that kills its host within a day. In contrast, death occurs slowly in the oral infection model, even though the pathogen is able to pass rapidly the multiple physical and immune barriers that protect the digestive tract (within 2–4 h, as evidenced with flies in which phagocytosis had been blocked to increase the sensitivity of detection; B. Kele, unpublished data). Thus, S. marcescens withstands passage through the cardia that guards the midgut entrance and expresses *Diptericin* as part of an immunological barrier. Subsequently, the bacteria are not contained by the peritrophic matrix that lines the midgut and halts most microorganisms. Finally, the bacteria escape the epithelium and cross the basal lamina to gain access to the body cavity, where their proliferation is kept in check by hemocytes.

### Serratia marcescens Db11 Resists the *imd*-Mediated Systemic Immune Response

In the septic injury model, Db11 kill wild-type and *imd* pathway mutant flies at the same speed. In contrast, LPS-defective strains are less virulent in wild-type flies but regain their full virulence when introduced in *imd* immunocompromised flies (Figure 1B and 1C). These data demonstrate that a major determinant of Db11′s virulence is its ability to resist the systemic immune response.

The resistance of Db11 could be caused by an ability to degrade AMPs or by a capacity to resist the attack of these antimicrobial peptides [16,27]. Since both S. marcescens Db1140 and 20C2 LPS-deficient mutants are sensitive to the systemic immune response, and since 20C2, in contrast to Db1140, is not impaired in protease secretion, it follows that resistance to AMP action is the major factor that determines virulence of Db11 in *Drosophila* after a septic injury. Previous experiments by Flyg and Xanthopoulos also support this conclusion. These authors determined that only Db1140 has a strongly attenuated virulence in comparison to its parent strain Db1121, which is also impaired in protease secretion but presumably has a normal O-antigen [27]. Taken together, these data indicate that the ability of S. marcescens Db11 to withstand AMP attack in vivo depends primarily on the presence of its O-antigen, in keeping with a similar phenomenon described for *Shigella* [39]. This may represent a novel mechanism of resistance to the action of AMPs [40]. It remains to be determined whether this effect is due to the structure of the LPS-O-antigen or whether the O-antigen is required to anchor or stabilize a putative microbial effector that would neutralize AMPs. This resistance mechanism, however, is not effective in the midgut environment.

###  Escape from the Intestinal Tract

One important question is that of the passage through the intestinal tract. We have been able to detect Db11 bacteria within midgut cells at a low frequency in wild-type flies during early infection. This rate increased significantly in *key* mutants. Interestingly, these bacteria were always observed inside vacuoles, in keeping with observations in human bladder cells that have internalized S. marcescens [41]. Some, or all, of these bacteria may be destroyed during this intracellular stage, as we could observe in *key* mutants many vacuoles containing debris of unknown origin. One possibility to account for the increased number of bacteria observed intracellularly in the *key* midgut epithelium is that many bacteria are prevented from reaching the midgut epithelium by the *imd*-dependent local immune response in wild-type flies. Alternatively, bacteria may access the wild-type or *key* gut epithelial cells at the same rate and be eliminated more efficiently in wild-type flies within the epithelial cells by the joint action of the *imd* response and a putative intracellular defense mechanism.

We have not yet observed by electron microscopy bacteria entering or exiting the intestinal epithelium. Thus, we cannot formally rule out that all intracellular bacteria are killed and that the actual passage occurs in between cells as opposed to an intracellular route. Presumably, the passage between cells would involve proteases to disrupt the junctions between adjacent cells. In this respect, we note that Db1140 bacteria that secrete a greatly reduced level of proteases are still able to traverse the intestinal barrier (Figure 7G) and can be detected intracellularly (Figure S6). This observation suggests that S. marcescens has an inherent ability to cross intact epithelia through the cells. We have failed to detect bacteria crossing the junctions that seals the epithelium at early stages of the infection. This mode of crossing may, however, be used at later times of the infection, when the integrity of the midgut appears to be severely affected. Consistent with this idea, the *imd* pathway–sensitive 20C2 bacteria are present in the hemolymph at a low level for 72 h, and then their number increases strongly in this compartment (Figure 2D). We propose that this increased passage results from an augmented “permeability” to bacteria of the intestinal epithelium that becomes compromised, possibly by secreted bacterial proteases.

### The Intestinal Immune Response as a Defense against Food-Borne Infections

Our experiments with Db11, and especially the *imd* pathway–sensitive strain 20C2 that regains wild-type virulence in *key* mutant flies, confirm and further document the importance of the local immune response in the midgut in the defense against intestinal infections (Figure 1) [16,17]. First, the *imd* pathway is activated in the midgut and, in contrast to previous studies [16,18], we find that the induction of the *imd* pathway is not limited to the cardia but extends to large portions of the midgut. Second, the bacterial load of *imd* pathway–sensitive 20C2 is lower than that of Db11 at 96 h of infection of the wild-type, whereas both bacterial titers are similar in a *key* mutant background (Figure 2E). Third, we could not detect any induction of the systemic immune response in the fat body, in contrast to other infection models [18,19]. Finally, the *key* mutant phenotype could be fully rescued by expressing a transgene only in the midgut.

While Db11 is resistant to the strong ubiquitous expression of *Diptericin* in the septic injury model (Figure S1A), it is sensitive to some degree to a strong expression of this AMP when present in the midgut (Figure 6F). However, since even under these nonphysiological conditions Diptericin is not able to provide full protection, the *imd* pathway must control the expression of other effectors of the gut local response. These additional defenses might include proteases, lysozymes, and nitrogen oxide production, as well as unidentified molecules. Even though we could not detect the induction of other AMP genes with our set of GFP reporter genes, we cannot exclude the possibility that *Cecropin* and *Attacin* are induced to some extent in our system, as described for other oral infection models [16,17]. The *imd* response may be potentiated or act in conjunction with the oxidative burst induced independently of the *imd* pathway by bacterial feeding [42,43]. Indeed, recent data indicate a partial sensitivity of flies in which the expression of the *Duox* gene, which mediates the oxidative burst, is decreased by transgenic RNAi (B. Kele, unpublished data). In this context, one can also note the presence of a catalase and of three SOD genes in the Db11 genome, which may mediate the resistance to this host defense (P. Giammarinaro and J. Ewbank, unpublished data). Our studies extend to *Drosophila* the concept that the epithelial response is an important and ancient aspect of host defense against infection [44–47].

### A Paramount Role for Phagocytosis in Host Defense against Natural Infections

Phagocytosis has often been described as playing an ancillary function in host defense against systemic bacterial infections in *Drosophila* [30,48]. This cellular process, however, plays a primordial role in our oral infection model, as it controls the proliferation of S. marcescens bacteria that have escaped from the alimentary canal. When phagocytosis is inhibited with latex beads, we observe septicemia, which is likely to cause the demise of the infected fly given the high bacterial load at the time of death. Indeed, a similarly high titer of bacteria is observed in the septic injury model. The importance of phagocytosis in the host defense against Db11 oral infections has been established in a separate study that used a mutant line defective for a novel phagocytic receptor gene, *eater*, where essentially the same results were obtained in terms of survival and Db11 bacterial growth [49].

The cellular response and the local response complement each other. Indeed, we observed that the effects of *imd* pathway and phagocytosis inactivation are additive (Figure 7B and 7C). This effect has been confirmed in mutants doubly defective for the IKKγ homolog, KEY, and for Eater (N. Nehme, unpublished data). Similarly, the protease-deficient Db1140 strain, which is also sensitive to the action of the *imd* pathway, could only proliferate in the hemolymph of *key* flies in which phagocytosis had been inactivated (Figure 7G).

### Decreased Virulence of S. marcescens in the Ingestion Model

A striking finding from the present study is that bacteria that have escaped into the body cavity of orally infected flies do not kill their host rapidly, in contrast to the septic injury model wherein flies succumb to infection by 100 bacteria in less than a day. S. marcescens is sensitive to phagocytosis in the ingestion model, while it is apparently not susceptible to it in the septic injury model. These findings suggest that S. marcescens does not express the same virulence program in both models, possibly as a result of its exposition to midgut defenses.

Consequently, bacteremia is unlikely to be the cause of lethality of wild-type flies fed on Db11, as the bacterial titer in the hemolymph is lower by two orders of magnitude than that measured in the septic injury model or when phagocytosis is blocked (compare Figure 2D to Figures 1D and 7E). Rather, we surmise that the death of flies is due to the severe degradation of the midgut epithelium. The progressive thinning of the midgut epithelium is reminiscent of that observed in C. elegans infections; however, in that model, the bacteria are unable to escape from the nematode intestine [22]. Flies or nematodes infected with Db1140, a strain displaying a reduced production of proteases, display an apparently normal structure of the intestinal epithelium and do not succumb to the infection, suggesting that bacterial proteases are involved in the attack of the intestinal epithelium.

### The Lack of Induction of the Systemic Response in the Oral Infection Model Supports a Model of Detection of Infection by Sensing Secreted PGN Fragments

The presence of *S. marcescens* in the digestive tract following oral infection does not induce a systemic immune response. One possibility is that S. marcescens does not proliferate enough in the gut to release small PGN fragments in quantities sufficient to overcome the immune-suppressive action of PGRP amidases in the gut and hemocoele that thus prevent the systemic activation of the *imd* pathway [20,50].

An unexpected finding is that S. marcescens bacteria present in the hemocoele after passage through the gut fail to elicit the systemic response, whereas they do when introduced by septic injury. This situation may be similar to that observed in *domino* mutant larvae, which lack hemocytes and harbor microorganisms in their hemolymph that also do not elicit the systemic immune response [48]. The lack of a systemic response to the presence of bacteria in the hemolymph is not due to the absence of a wounding response in this model of infection, because a clean injury performed on flies that have ingested Db11 did not induce the *imd* pathway any more than in flies fed on a sugar solution (unpublished data). In contrast, a marked systemic immune response was observed following a block of phagocytosis (Figure 8C). One supposition is that bacteria in the hemolymph fail to grow and divide actively and thus do not release PGN fragments. This hypothesis would explain why the net increase in bacterial number in the hemolymph is mostly due to bacterial passage from the gut (Figure 8B). However, to account for the stimulation of the systemic response when phagocytosis is blocked, one would have to hypothesize that phagocytosis somehow inhibits bacterial growth and division; this inhibition would be relieved upon ingestion of latex beads. We have so far failed to obtain any direct evidence in support of Db11 being in a dormancy state [51,52]. Alternatively, Db11 bacteria divide actively in the hemolymph and are phagocytosed at a rate that approximately equals that of divisions, since the net increase of the bacterial number in this compartment appears to be mostly due to bacteria transferred from the gut (Figure 8). The continuous passage of bacteria from the intestine would compensate the bacterial loss due to phagocytosis. In the absence of a supply of fresh bacteria from the intestine, the bacteria are cleared from the hemolymph, as observed in the gentamicin experiments. We therefore propose that phagocytosis of growing and dividing bacteria keeps the levels of PGN fragments below the threshold of detection.

In larvae, it has been proposed that hemocytes are required to signal the presence of bacteria to the fat body to trigger the systemic immune response, either by emitting a cytokine or by releasing PGN fragments after phagocytosis [18,53]. This model is appealing because the PGN layer of the Gram-negative cell wall is not directly accessible and is buried under the outer membrane and a LPS shell, and thus some sort of cellular processing might be required to uncover the PGN polymers. Our data, however, argue against such a model in adults, since we observe a systemic response only when phagocytosis is blocked. Rather, our results are best accounted for by the release of short PGN fragments during bacterial growth and proliferation. Furthermore, in a septic injury model, we observed a sustained activation of the *imd* pathway in flies in which phagocytosis was impaired by the prior injection of latex beads (N. Nehme, unpublished data).

### Perspectives

The immunity of mucosal surfaces, especially that of the intestinal epithelium, is the focus of intense scrutiny [54]. The human digestive tract, however, is complex since it harbors more than 400 distinct microbial species and is protected by both innate and adaptive immune responses [55]. In contrast, *Drosophila* provides a simple and powerful model that allows the dissection of the innate immune responses in the digestive tract. In addition, these studies can be performed at the whole organism level, as exemplified by the possibility of investigating phagocytosis [49] as a complement to intestinal defenses. Because it is able to cross the fly intestinal barrier, and because it is resistant to some of the host immune responses, S. marcescens Db11 constitutes an attractive model for the in vivo study of enteric pathogenesis. Interestingly, the treatment of cancer patients by chemotherapy leads to neutropenia and associated bacterial translocation and bacteremia, a striking parallel to the mechanism we describe in phagocytosis-deficient flies infected orally with S. marcescens. Further, this microorganism is amenable to genetic analysis and manipulation [56] and its genome has recently been sequenced (http://www.sanger.ac.uk/Projects/S_marcescens/). The stage is now set for a thorough investigation of the host–pathogen relationships between *Drosophila* and S. marcescens from the vantage of both the fly and that of the bacterium.

## Materials and Methods

### Bacterial strains and culture.

The nonpigmented S. marcescens strain Db10 was isolated originally from a moribund fly (Db is for *Drosophila* bacterium); Db11 is a spontaneous streptomycin-resistant mutant of Db10 [25]. Db1121 was derived from Db11 following two rounds of chemical mutagenesis and selection for decreased secretion of proteases. Db1140 is a spontaneous mutant derived from Db1121 that is resistant to phage ΦJ [27].

The Db11 miniTn5Cm insertion mutant 20C2 was described in Kurz et al. [22]. Since its original isolation, the locus affected in 20C2 has been better characterized thanks to whole genome sequencing. The insertion site is at genomic position 914398 and disrupts SMA0873 involved in O-antigen biosynthesis (http://www.sanger.ac.uk/Projects/S_marcescens/). The O-antigen biosynthesis operon spans genes SMA0868 to SMA0879. Other mutants in this operon have been isolated from a library of Tn5 insertion mutants generated in Db10 bacteria (Db11 is a streptomycin-resistant strain derived from Db10; Db10 behaves as Db11 in oral and septic injury infection assays (N. Nehme, unpublished data; [25])). These mutants correspond to transposon insertions into SMA082, SMA0873, and SMA0876. GFP or DsRed derivatives of Db11were obtained by transformation with plasmids pUFR-GFP (ampicillin and gentamicin resistance) or pEP933 (tetracyclin and gentamicin resistance), respectively. The DsRed- and GFP-labelled S. marcescens transformants behaved as their cognate strain in both *Drosophila* infection models. Strains were grown in LB (Luria Bertani medium) at 37 °C. When required, antibiotics were added at the following concentrations: ampicillin, 100 μg/ml (Db11GFP); streptomycin, 100 μg/ml; chloramphenicol, 30 μg/ml; and gentamicin, 10 μg/ml.

### Fly strains.

Stocks were raised on standard corneal-agar medium at 25 °C. *cn bw* flies were used as wild-type for most of the experiments since *key^1^* and *Dif^1^* mutants were generated in this background [57]. *Dredd*, *FADD*, *Relish*, and *PGRP-LE* mutant strains were respectively described in [58–61]. The A5001 strain is the wild-type strain that was used to generate the *PGRP-LC* mutant strain [6]. Where indicated, we used an Oregon-R stock as wild-type control. The *UAS-Diptericin* line has been described previously [4]. The *UAS-key* and *pTEP-GFP* lines were kind gifts of Sophie Rutschmann and Daniel Doucet, respectively. *pTEP-GFP* line was generated using the promoter of the *TEP1* gene fused to *GFP*; this construct is specifically expressed in hemocytes (unpublished data). All the mutant lines are described on the Flybase Web site (http://flybase.bio.indiana.edu/). The NP1 and NP3084 lines were selected in a screen of enhancer trap Gal4 lines expressed in embryonic and/or larval gut tissues and available from the *Drosophila* Genetic Resource at the National Institute of Genetics (Shizuoka, Japan; http://www.shigen.nig.ac.jp/fly/nigfly/). These strains were selected for their strong and specific expression in the midgut of adult flies. No expression has been observed in the malpighian tubules or other surrounding tissues. The *key* rescue lines were constructed by standard genetic crosses.

### Survival experiments.

Survival experiments were performed as previously described [62]. Briefly, batches of 20–25 wild-type and mutant flies were challenged by septic injury using a needle previously dipped in a concentrated solution of E. coli. As regards S. marcescens, an overnight culture was diluted to an optical density at 600 nm (OD_600_) of 0.1 in LB. This leads to the inoculation of 50–100 bacteria per fly, as checked by performing colony counts on flies crushed right after septic injury. The vials containing the challenged flies were then put in an incubator at the desired temperature and the surviving flies counted every few hours. Each experiment shown is representative of at least three independent experiments. Statistical tests were performed using the Log Rank test within Prism software.

### Fly infection by feeding.

Batches of 20–25 adult wild-type and mutant female flies were used in these experiments. The food solution containing bacteria was prepared from a culture grown exponentially at 37 °C to OD_600_ = 1. This culture was diluted with a sterile 50-mM sucrose solution to a final OD_600_ = 0.1. For Db1140 infections, the bacteria were collected by centrifugation and resuspended directly in the 50-mM sucrose solution. A pile of folded papers (Tork) was placed in the bottom of medium-sized fly culture tubes and soaked with about 2.5 ml of the contaminated sucrose solution. The flies were then transferred to these vials and fed continuously on this solution. Surviving flies were usually counted twice a day. Most experiments were performed at 25 °C, except for experiments involving *Gal4* drivers, which were conducted at 29 °C.

### Injection of latex beads.

Sixty-nine nanoliters of 4-fold concentrated Surfactant-Free Red CML Latex beads (0.30 μm-diameter polystyrene beads; Interfacial Dynamics Corp) were injected into recipient flies to block phagocytosis, as previously described [63]. The effectiveness of the procedure was checked by testing the phagocytosis of FITC-labeled E. coli [30].

### Growth of S. marcescens in vivo.

*Whole flies count*. Flies were infected in batches of 20 with S. marcescens Db11-GFP or 20C2-GFP. Flies were crushed in 0.5 ml of LB medium at various times after infection using a micropestle, and the homogenate was serially diluted in LB medium. The number of colony-forming units (CFU) was determined through growth overnight at 37 °C on LB agar with the appropriate antibiotics.

*Hemolymph count.* Hemolymph was collected from batches of 20 flies by pricking with an empty capillary mounted on a Nanoject II (Drumond Scientific). The hemolymph was collected in sterile PBS on ice, serially diluted, and plated with the appropriate antibiotics.

*Intestinal count.* The experiment was done as described above for whole flies except that the intestines of 20 infected flies were dissected in sterile PBS and collected in PBS on ice.

### Quantitative reverse-transcription PCR.

This analysis was done as previously described [9].

### Fluorescent microscopy and imaging.

For the images in Figure 2A, intestines were dissected in PBS and immediately observed using a Zeiss SteREO Lumar.V12 dissection microscope equipped with an AxioCam camera and AxioVision 4.1 software.

For Apotome microscopy, intestines were dissected in PBS, mounted in Vectashield, and observed immediately using a Zeiss Axiovert 200 inverted microscope equipped with an AxioCam camera and AxioVision 4.1 software. Optical sections through the fluorescent sample were taken using the Apotome fringe projection system. To visualize GFP, a FITC filter set was used, whereas the rhodamine filter set was employed for DsRed.

For confocal microscopy, dissected guts were fixed 30 min in 4% paraformaldehyde (PFA) and stained 1 h by 10 μM FITC-labeled phalloidin (Fluka) in PBS + 0.1% Triton X100 for 2 h. Guts were observed under an inverted Zeiss Axiovert 100 M microscope equipped with the LSM510 laser scanning confocal module Images were processed with LSM510 (version 2.5) and ImageJ (version 1.37h) software.

### Transmission electron microscopy.

Fly midguts were dissected in phosphate buffer 0.1 M (pH 7.2) and fixed with 4% glutaraldehyde for 30 min at room temperature. Samples were postfixed for 4 h with 1% osmium tetroxide in the same buffer at 4 °C, rinsed, dehydrated through a graded ethanol series, and embedded in Epon/araldite resin. Ultra-thin sections were contrasted with uranyl acetate and lead citrate. Sections were observed at 60 kV on a Hitachi 7500 transmission electron microscope.

### Frozen sections and immunohistochemistry.

Midguts of adult flies were dissected, fixed for 30 min at room temperature in 4% PFA in phosphate buffer (pH 7.2), and infiltrated overnight with 0.44 M phosphate-buffered sucrose. After being rapidly frozen in 0.22 M phosphate-buffered sucrose with 7.5% gelatine, 8-μm-thick sections were cut on a Leica CM3050S cryostat.

Sections were then blocked for 30 min in PBS + 2% BSA and incubated overnight with the anti-α-spectrin monoclonal antibody (antibody 3A9, obtained from the Developmental Studies Hybridoma Bank) at a 1:10 dilution in PBS + 0.2% BSA. Sections were then incubated for 1 h with goat anti-mouse secondary antibodies conjugated to Alexa488 (Molecular Probes).

Finally, sections were incubated for 15 min at room temperature in a 5 μg/ml DAPI solution in PBS.

### Staining of β-galactosidase activity.

Tissues were dissected and fixed in a glutaraldehyde 1% solution for 10–15 min, and stained in a 30 μl/ml X-gal stock solution (5% in DMF).

### Cytochalasin D injection experiments.

Cytochalasin D was dissolved in DMSO to make a 1 mg/ml concentrated solution, which was diluted in PBS to a 20 μg/ml solution. One hundred nanoliters of this solution was injected in each treated fly. Mock-injected flies were injected with a PBS solution containing 2% DMSO. The efficiency of the treatment on phagocytosis inhibition was checked as described [30].

## Supporting Information

Figure S1 *Diptericin* Overexpression Confers a Slight Resistance to *S. marcescens* Infection in the Ingestion but Not in the Septic Injury Model (A) Wild-type, mutants for IMD pathway (*key*) or *Toll* pathway (*Dif*), and *Diptericin* overexpressing flies (Hsp-Dipt: flies carrying *UAS-Diptericin* and the strong *hsp-Gal4* driver transgenes were placed at 37 °C for two 30-min periods) die at the same rate after a Db11 challenge (septic injury).
(B) Wild-type flies that overexpress a *UAS-Diptericin* transgene in the midgut under the control of the *NP3084* driver are somewhat more resistant to S. marcescens intestinal infections.
(21 KB PDF)

Figure S2 Death Rate of Infected Flies Varies with the Bacterial Load and Temperature Wild-type (wt) and *key* flies were continuously fed on different concentrations of Db11 (OD = 0.1 or 0.5) at 29 °C, and survival was monitored daily and expressed in % of surviving flies. At 29 °C, wt and *key* flies die, respectively, 2 d and 1 d earlier than at 25 °C. Flies fed on higher concentrations of bacteria succumb earlier to the infection.
(19 KB PDF)

Figure S3 Db11 Penetrates the Invaginations of Copper Cells Confocal optical sections through the midgut of insects feeding on Db11-DsRed, after fixation and FITC-labeled phalloidin staining. Copper cells are found in a middle domain of the midgut and are responsible for the acidification of this portion of the digestive tract [33].
(A) 24 h after Db11-DsRed infection, the typical apical invaginations of the copper cells are shown by arrows.
(B) 48 h after Db11-DsRed infection, the invagination of a copper cell is filled with bacteria, suggesting that the integrity of the peritrophic membrane is affected. Two transverse sections were made through the copper cell in the inserts (1) and (2). Note the canal (along the right hand side of axis 2) that delivers acidic secretions into the gut lumen and that allows the entrance of Db11 within the invaginations.
(73 KB PDF)

Figure S4 Bacteria That Have Escaped from the Midgut Colonize Muscle and Tracheal Tissues The left-hand panel displays a DIC picture of the surface of the intestine; the central panel shows GFP fluorescence emitted by Db11-GFP bacteria 48 h after infection. The right-hand panel shows the superposition of both pictures. Bacteria are found mostly in the intestinal radial muscles (arrow) and in tracheoles (arrowheads).
(134 KB PDF)

Figure S5 Bacteria Can Be Seen Passing through and Inside Gut Cells after 48 h of Infection with Db11-DsRed at Higher Concentration (OD = 0.5) Confocal optical sections through the cardia (A, B, D) or the anterior midgut (C) of insects feeding on Db11-DsRed, after fixation and FITC-labeled phalloidin staining.
(A) Bacteria are observed inside the inner lumen of the cardia, after 72 h of infection (OD = 0.1). The inner lumen is the prolongation of the foregut in the digestive tract.
(B–D) After 48 h of infection at higher concentration (OD = 0.5), bacteria are also seen in the external lumen of the cardia ([B], inserts 1 and 2) and appear to pass through the cell layer that separates the inner lumen from the external one (D). Bacteria seem to pass through and between gut cells in the anterior midgut (C).
(78 KB PDF)

Figure S6 Db1140 Can Also Go through Gut Epithelium, but without Affecting the Integrity of the Gut Time series of tubulin-GFP-expressing flies feeding on Db1140-DsRed bacteria. Bacteria are sometimes observed within epithelial midgut cells (arrows). In addition, the structure of the gut appears to be preserved during the course of the infection.
(27 KB PDF)

Figure S7 *imd* Pathway Mutant Flies Are Sensitive to S. marcescens Oral Infections Mutants fly lines for genes of the *imd-*pathway were fed on S. marcescens Db11 (A, C) or 20C2 (B) and their survival monitored. These mutant flies succumb earlier than wild-type controls. *PGRP-LC^ΔE12^* null mutants appear to be somewhat more resistant than other *imd* pathway mutants. While *PGRP-LE* mutants behave as wild-type flies to Db11 ingestion, *PGRP-LE ; PGRP-LC* double mutants do not display a stronger phenotype than *PGRP-LC* mutants. A5001 is the wild-type control strain for *PGRP-LC*.
(37 KB PDF)

Figure S8 Flies Injected with Cytochalasin D Are More Susceptible to Db11 Oral Infections Flies challenged by a Db11 septic injury (si) die at the same rate whether they have or have not been pre-injected with cytochalasin. Flies injected with cytochalasin die earlier than nontreated control flies that are just starting to succumb when this survival experiment was stopped. Cytochalasin is not toxic to the flies since they survive normally, like PBS-injected flies. DMSO was used to dilute the cytochalasin and is not toxic at the concentration used. Oregon flies were used in this experiment.
(27 KB PDF)

Table S1 Expression Patterns of AMP Reporter Genes during Db11 Oral Infections (31 KB DOC)

Table S2 Bacterial Proliferation in the Hemolymph of Latex Bead–Treated Flies in the Absence of a Gut Bacterial Supply Counts of bacteria present in the intestine or hemolymph from 20 uninjected or latex bead–injected (LXB) wild-type Oregon flies after 24 h of Db11 feeding followed by 24 h feeding on gentamicin-sucrose solution.
(31 KB DOC)

## Abbreviations

AMP: antimicrobial peptide
d: day
DIF: Dorsal-related immunity factor
GNBP: Gram-negative binding protein
*imd*: *immune deficiency*
*key*: *kenny*
LPS: lipopolysaccharide
PGN: peptidoglycan
PGRP: Peptidoglycan recognition protein
ROS: reactive oxygen species
