# Supplementary material for: A Model of Bacterial Intestinal Infections in Drosophila melanogaster
Source: PLoS Pathog. 2007 Nov 23;3(11):e173. doi: 10.1371/journal.ppat.0030173 (PMC2094306; doi:10.1371/journal.ppat.0030173)
Supplement: Supplementary file 1 [file ppat.0030173.sg001.pdf]

A

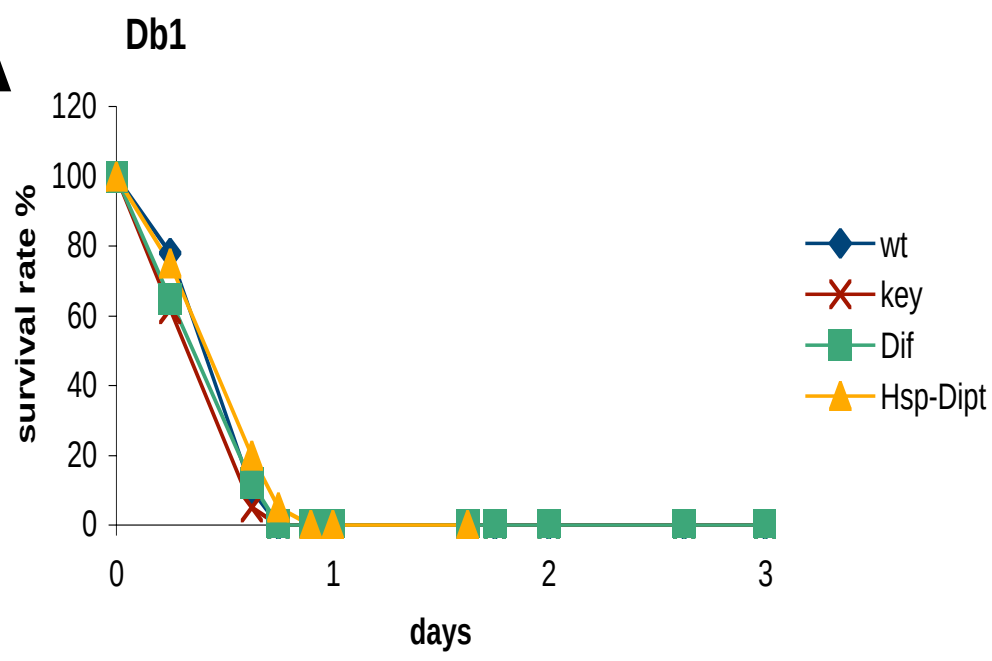
Db1
120
100
80
60
40
20
0
survival rate %
0
1
2
3
days
wt
key
Dif
Hsp-Dipt

B

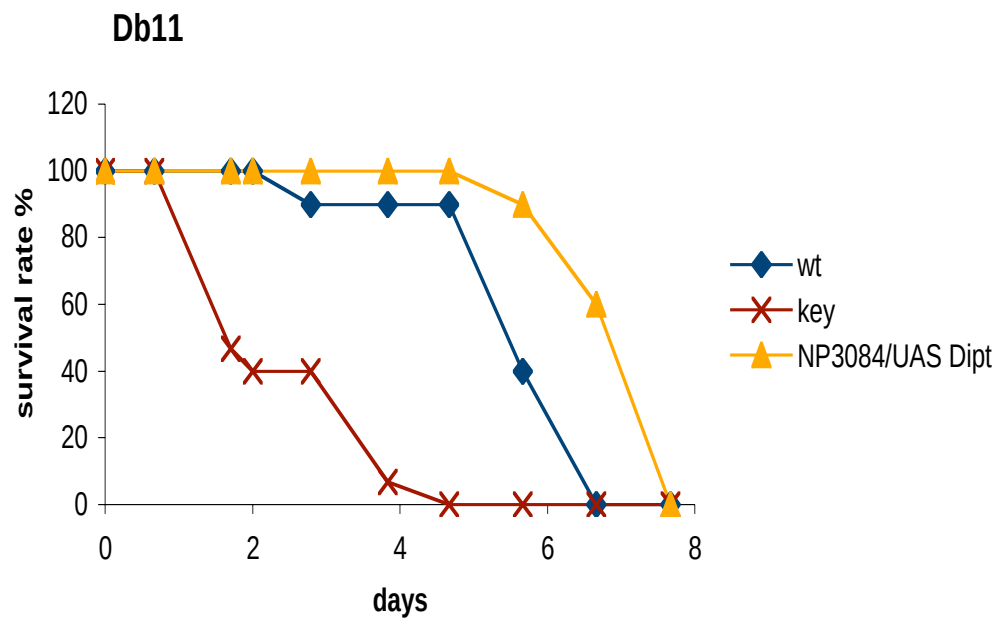
Db11
120
100
80
60
40
20
0
survival rate %
0
2
4
6
8
days
wt
key
NP3084/UAS Dipt
