# Supplementary material for: A Model of Bacterial Intestinal Infections in Drosophila melanogaster
Source: PLoS Pathog. 2007 Nov 23;3(11):e173. doi: 10.1371/journal.ppat.0030173 (PMC2094306; doi:10.1371/journal.ppat.0030173)
Supplement: Supplementary file 2 [file ppat.0030173.sg002.pdf]

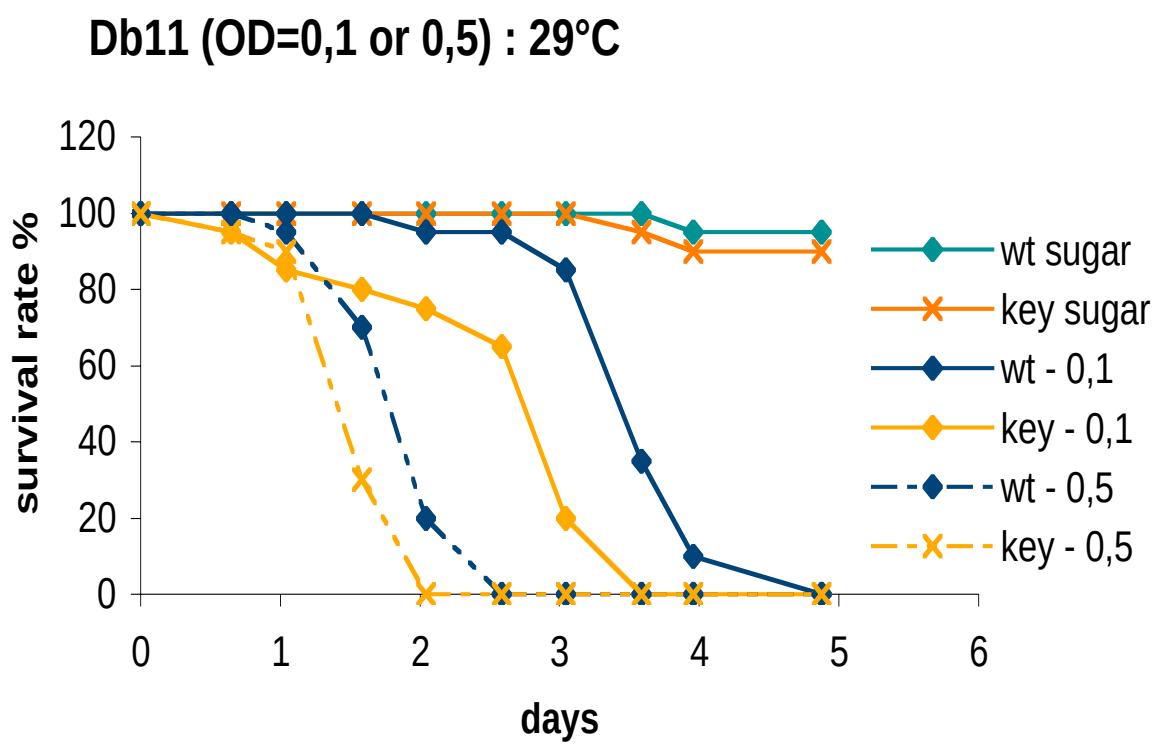

Db11 (OD=0,1 or 0,5) : 29°C
survival rate %
120
100
80
60
40
20
0
0
1
2
3
4
5
6
days
wt sugar
key sugar
wt - 0,1
key - 0,1
wt - 0,5
key - 0,5
