# Supplementary material for: A Model of Bacterial Intestinal Infections in Drosophila melanogaster
Source: PLoS Pathog. 2007 Nov 23;3(11):e173. doi: 10.1371/journal.ppat.0030173 (PMC2094306; doi:10.1371/journal.ppat.0030173)
Supplement: Supplementary file 5 [file ppat.0030173.sg005.pdf]

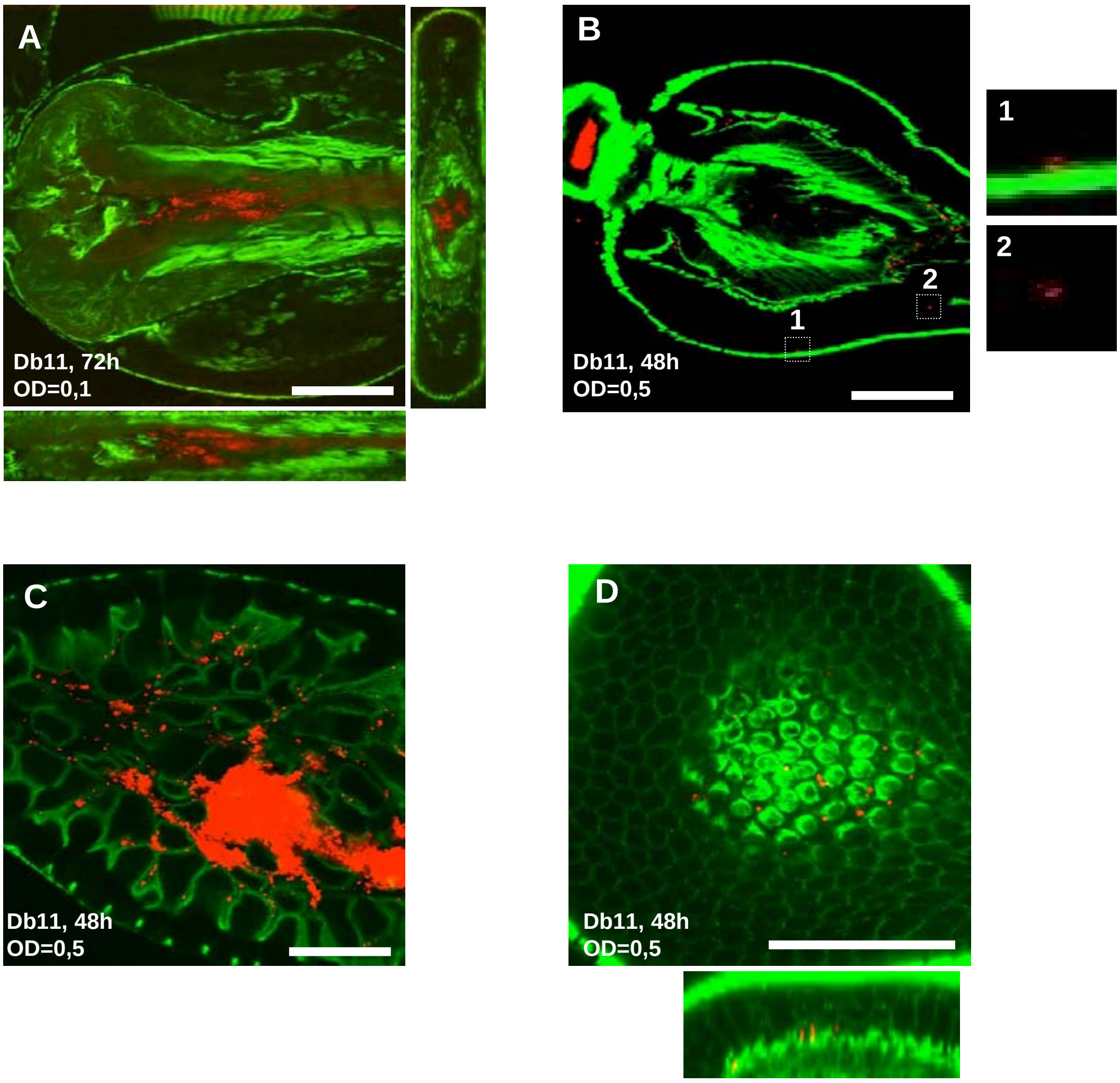

A
Db11, 72h
OD=0,1
B
1
2
Db11, 48h
OD=0,5
C
Db11, 48h
OD=0,5
D
Db11, 48h
OD=0,5
