# Supplementary material for: A Model of Bacterial Intestinal Infections in Drosophila melanogaster
Source: PLoS Pathog. 2007 Nov 23;3(11):e173. doi: 10.1371/journal.ppat.0030173 (PMC2094306; doi:10.1371/journal.ppat.0030173)
Supplement: Supplementary file 6 [file ppat.0030173.sg006.pdf]

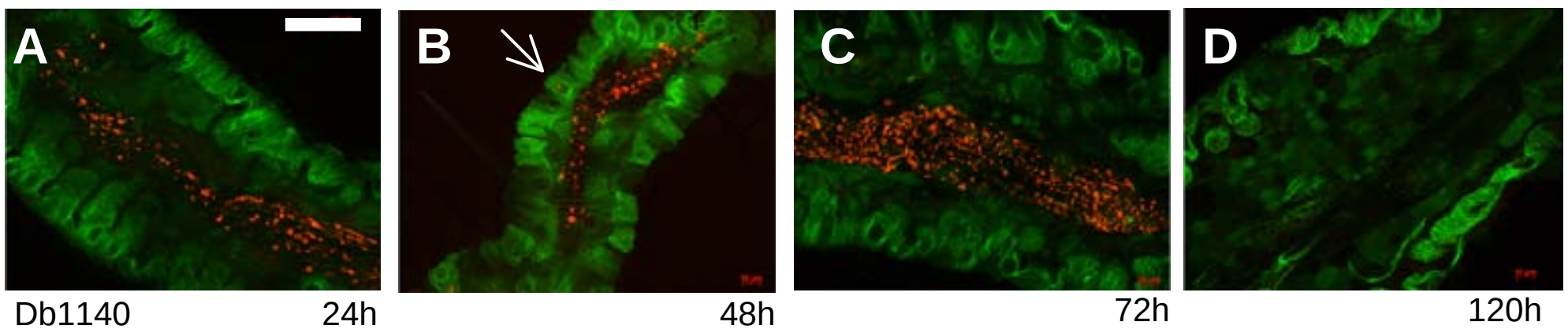
A
B
C
D
Db1140
24h
48h
72h
120h
