# Supplementary material for: A Model of Bacterial Intestinal Infections in Drosophila melanogaster
Source: PLoS Pathog. 2007 Nov 23;3(11):e173. doi: 10.1371/journal.ppat.0030173 (PMC2094306; doi:10.1371/journal.ppat.0030173)
Supplement: Supplementary file 7 [file ppat.0030173.sg007.pdf]

A

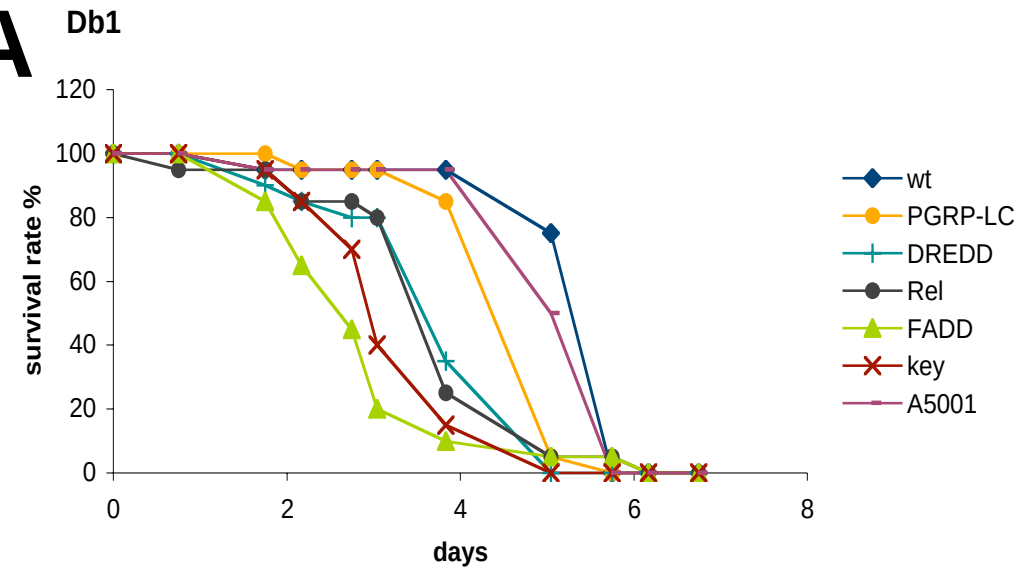

Db1
survival rate %
120
100
80
60
40
20
0
0
2
4
6
8
days
wt
PGRP-LC
DREDD
Rel
FADD
key
A5001


B

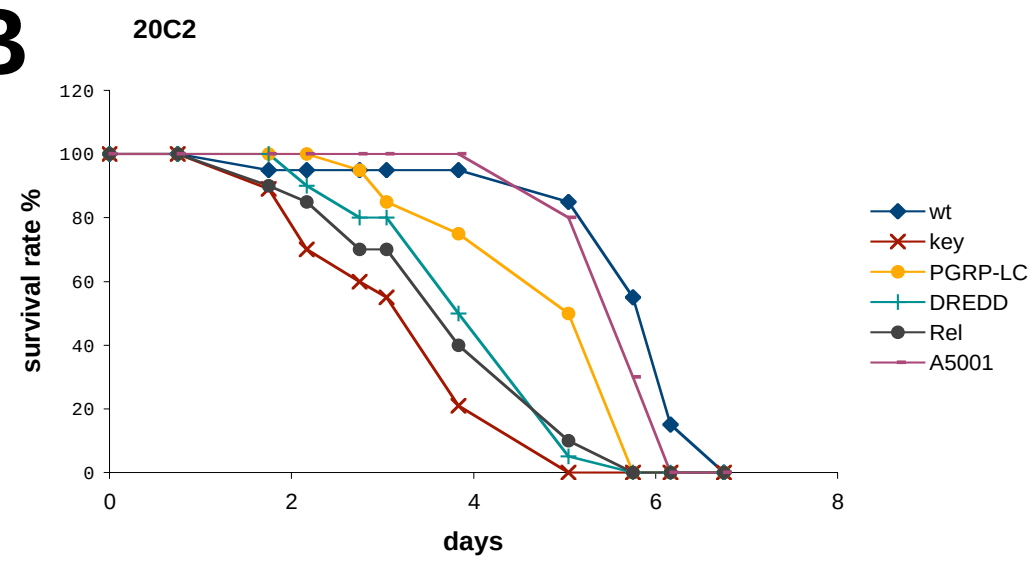

20C2
survival rate %
120
100
80
60
40
20
0
0
2
4
6
8
days
wt
key
PGRP-LC
DREDD
Rel
A5001


C

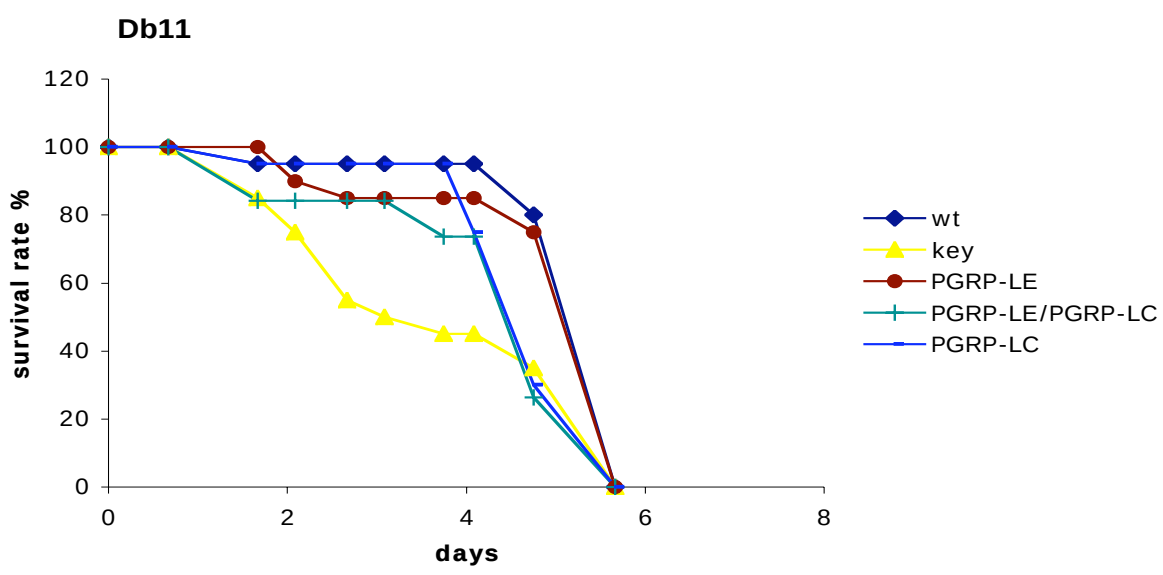

Db11
survival rate %
120
100
80
60
40
20
0
0
2
4
6
8
days
wt
key
PGRP-LE
PGRP-LE/PGRP-LC
PGRP-LC
