# Supplementary material for: A Model of Bacterial Intestinal Infections in Drosophila melanogaster
Source: PLoS Pathog. 2007 Nov 23;3(11):e173. doi: 10.1371/journal.ppat.0030173 (PMC2094306; doi:10.1371/journal.ppat.0030173)
Supplement: Supplementary file 8 [file ppat.0030173.sg008.pdf]

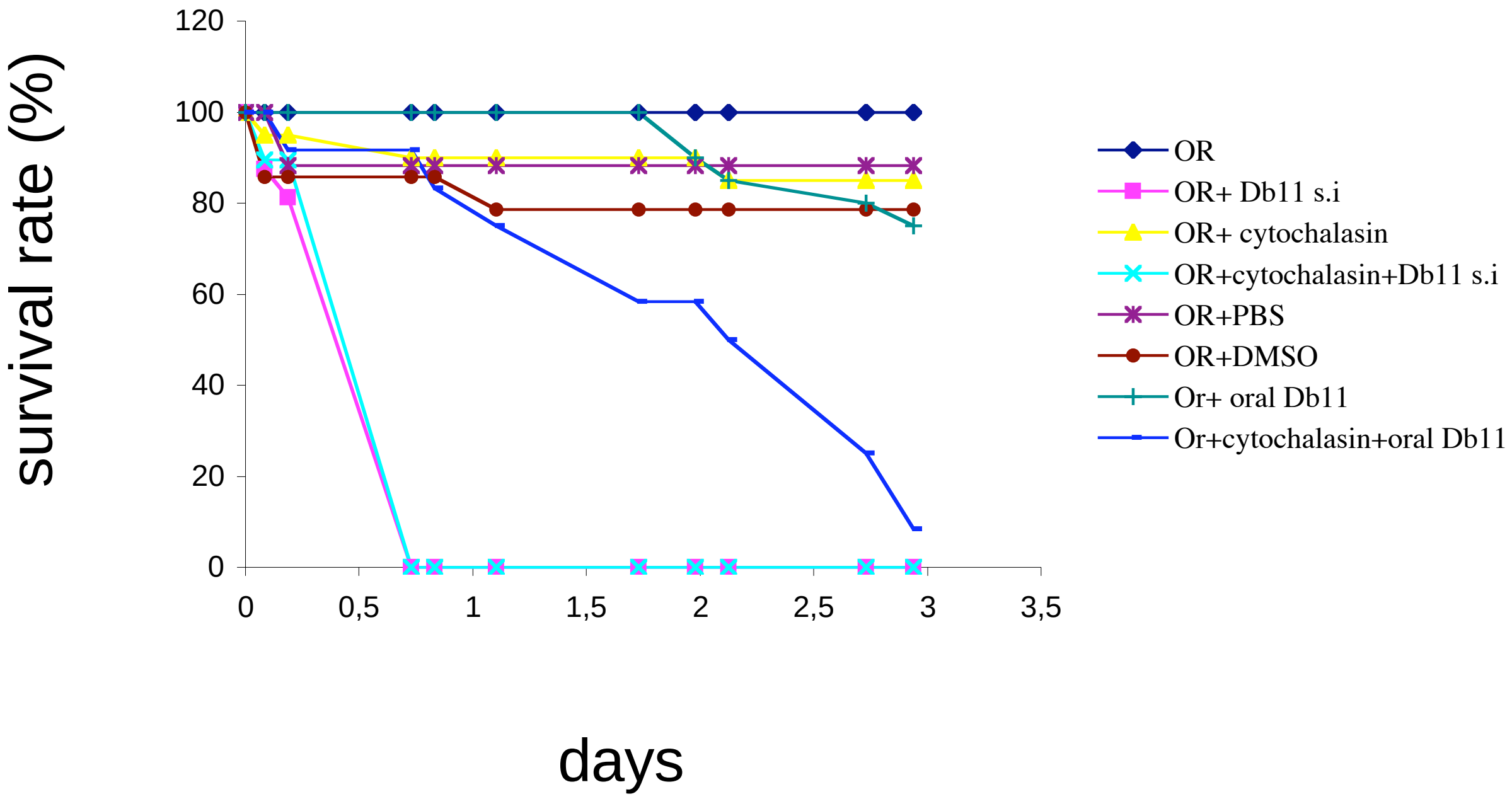

survival rate (%)
120
100
80
60
40
20
0
0
0,5
1
1,5
2
2,5
3
3,5
days
OR
OR+ Db11 s.i
OR+ cytochalasin
OR+cytochalasin+Db11 s.i
OR+PBS
OR+DMSO
Or+ oral Db11
Or+cytochalasin+oral Db11
