# Supplementary material for: A Model of Bacterial Intestinal Infections in Drosophila melanogaster
Source: PLoS Pathog. 2007 Nov 23;3(11):e173. doi: 10.1371/journal.ppat.0030173 (PMC2094306; doi:10.1371/journal.ppat.0030173)
Supplement: Supplementary file 9 [file ppat.0030173.st001.doc]

labellum

cephalic fat body

labellum

cephalic fat body

labellum

Metchnikovin-GFP

tracheal system

tracheal system

tracheal system

Drosomycin-GFP

not expressed

not expressed

not expressed

Defensin-GFP

intestine

intestine

intestine

Diptericin-GFP

not expressed

not expressed

not expressed

Cecropin-GFP

not expressed

not expressed

epidermis

Attacin-GFP

wings

wings

wings

Drosocin-GFP

72h p.i

48h p.i

24h p.i*

- p. i. : post infection
