# Supplementary material for: A Model of Bacterial Intestinal Infections in Drosophila melanogaster
Source: PLoS Pathog. 2007 Nov 23;3(11):e173. doi: 10.1371/journal.ppat.0030173 (PMC2094306; doi:10.1371/journal.ppat.0030173)
Supplement: Supplementary file 10 [file ppat.0030173.st002.doc]

|  | Intestine | | | Hemolymph | | |
| --- | --- | --- | --- | --- | --- | --- |
| Time^a^ | Control^b^ | wt | wt + LXB | Control | wt | wt + LXB |
| 0 h | 27 E 04 | 27 E 04 | 24 E 05 | 160 | 160 | 3260 |
| 24 h | 33 E 04 | 36 | 5 E 04^c^ | 260 | 11 | 22080 |

^a^ Period of feeding on 500 µg/ml gentamicin

^b^ Control : continuous feeding on Db11 without gentamicin feeding

^c^ The high number of bacteria recovered in the intestine of latex bead-injected, gentamicin-treated flies is not due to a failure of the antibiotics treatment but most likely to the adsorption of bacteria present in the body cavity to the gut. Indeed, flies infected by septic injury also display a high number of bacteria associated with the gut, even though those flies have not been orally infected. We have checked by microscopy that few viable bacteria were present in the gut lumen of such flies.

Qualitatively similar results have been obtained with a 4 hour period of Db11 feeding.
